# Monolayer-Defined Flat Colloidal PbSe Quantum Dots in Extreme Confinement

**DOI:** 10.1021/acs.nanolett.5c02957

**Published:** 2025-07-28

**Authors:** Leon Biesterfeld, Huu Thoai Ngo, Ahmed Addad, Dominik A. Rudolph, Wolfgang Leis, Michael Seitz, Gang Ji, Bruno Grandidier, Christophe Delerue, Jannika Lauth, Louis Biadala

**Affiliations:** † Cluster of Excellence PhoenixD (Photonics, Optics, and Engineering − Innovation Across Disciplines), Welfengarten 1A, D-30167 Hannover, Germany; ‡ Institute of Physical and Theoretical Chemistry, Eberhard Karls University of Tübingen, Auf der Morgenstelle 18, D-72076 Tübingen, Germany; § Institute of Physical Chemistry and Electrochemistry, Leibniz University Hannover, Callinstr. 3A, D-30167 Hannover, Germany; ∥ Université de Lille, CNRS, Centrale Lille, Université Polytechnique Hauts-de-France, Junia-ISEN, UMR 8520-IEMN, F-59000 Lille, France; ⊥ Université Lille, CNRS, INRAE, Centrale Lille, UMR 8207-UMET-Unité Matériaux et Transformations, F-59000 Lille, France; # Institute of Inorganic Chemistry, Eberhard Karls University of Tübingen, Auf der Morgenstelle 18, D-72076 Tübingen, Germany

**Keywords:** lead selenide, colloidal 2D nanocrystals, scanning tunneling spectroscopy, quantum confinement, dimensionality, near-infrared, monolayer

## Abstract

Colloidal 2D PbX (X = S, Se, Te) nanocrystals are innovative materials pushing the boundaries of quantum confinement by combining crystal thicknesses down to a monolayer with additional confinement in the lateral dimension. These flat PbSe quantum dots (fQDs) exhibit telecommunication band photoluminescence (1.43–0.83 eV), which is highly interesting for fiber optic information processing. With scanning tunneling microscopy/spectroscopy (STM/STS), we probe single-layer-defined fQD populations down to one monolayer, showing an in-gap state free QD-like density of states in excellent agreement with theoretical tight-binding (TB) calculations. Cryogenic ensemble spectra match STS/STM and TB calculations and exhibit the contribution of mono-, bi-, and trilayers to the photoluminescence. Comparing the electronic band gaps with the optical ones, we derive exciton binding energies as high as 600 meV for PbSe monolayers. Our results allow for a target-oriented synthesis of a **new class** of QDs with record binding energies and precisely tailored optical properties at technologically relevant wavelengths.

## Graphical abstract

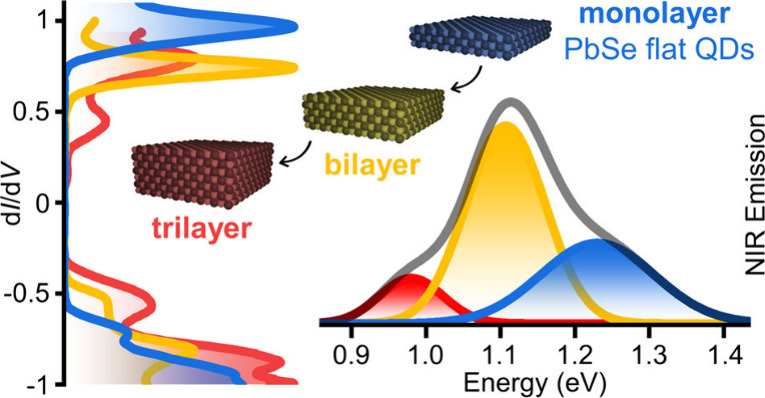

Controlling the anisotropic growth of semiconductors at the nanoscale is key for tuning the degree of freedom of charge carriers, i.e., by dimensionality,^1–3^ and for the development of quantum technologies.^4,5^ Among the available growth techniques, colloidal synthesis stands out, as it allows for a growth-by-design by thorough control over the size and the shape^6–9^ of semiconductor nanocrystals (NCs), which enables fine-tuning of their (photo)­physics and optoelectronic properties.^10,11^ A striking example is the growth of colloidal 2D CdSe nanoplatelets (NPLs), for which the thickness is controlled at the atomic layer scale,^12^ while the in-plane dimensions can be tuned over hundreds of nanometers.^13^ The strong quantum confinement in the vertical dimension dominates the optical properties of the material, resulting in rapidly decaying (<10 ns at room temperature (RT)) PL associated with narrow line widths below 40 meV and exciton binding energies of ∼200 meV.^12,14–16^ At the same time, the lateral confinement in 2D CdSe enables a fine-tuning of the exciton energy^17^ by reducing the length or width of the NPLs down to and below the exciton Bohr radius. However, the optical properties of CdSe are typically limited to visible wavelengths, and to reach the telecommunication range, smaller band gap materials such as lead chalcogenides (PbX (X = S, Se, Te)) are required.^18–20^ Notwithstanding their high potential as classical or quantum emitters for applications in fiber optics and photonics,^21,22^ PbX NCs stand out for their remarkable physical properties with large exciton Bohr radii^23,24^ and high dielectric constants.^25^ Furthermore, PbX nanocrystals (NCs) are predicted to exhibit intriguing properties when extreme quantum confinement is reached. For example, PbSe in its monolayer (1ML) form could be a topological crystalline insulator^26,27^ and a suitable material platform for defect engineering.^28^ Notably, Ekuma reported on the effect of vacancy defects on the electronic and optical properties of monolayer PbSe and found that defect-induced states predominantly reside outside the band gap, which renders monolayer PbSe a rather defect-tolerant material.^28^ Scarce experimental realization of monolayer-defined PbSe islands by molecular beam epitaxy has demonstrated the strong influence of the van der Waals substrate (MoS_2_ ^29^ or VSe_2_ ^30^) on the growth process and the resulting material properties, caused by the induced strain between the crystal lattices of PbSe and the van der Waals substrate.^29,30^ Here, we define a PbSe monolayer (1ML) according to the unit cell of cubic PbSe, namely, 0.6 nm (Pb-to-Pb distance), which consists of three atomic planes of PbSe. Recent colloidal synthesis protocols for cubic rock salt crystal-structured anisotropic PbS, PbSe, and PbTe NCs^31–34^ allow to circumvent substrate interactions and are crucial for further harnessing the extreme quantum confinement in substrate-free PbX materials.

Flat PbSe NCs studied here are referred to as fQDs in the following and exhibit efficient PL (up to 61% quantum yield) in the NIR between 1.43 and 0.83 eV (860–1510 nm).^31,33^ While highly promising, fQDs have been mostly studied with ensemble optical spectroscopic techniques, which provide average excitonic properties, but prevent comprehensive knowledge of the electronic properties, such as the dimensionality, the presence of electronic trap states in the gap, and exciton binding energies in correlation with structural properties (3D dimensions and associated size dispersion). In contrast, scanning tunneling microscopy (STM) allows for the determination of the single-particle morphology, and scanning tunneling spectroscopy (STS) enables probing excitation spectra of individual NCs, practically without any selection rules.^35^ For colloidal lead chalcogenide NCs, STM/S studies have focused on spherical 0D PbSe QDs,^36,37^ PbSe/PbS core–shell QDs,^38^ “molecular” aggregates of QDs,^36^ and their epitaxially fused superlattices up to now.^39–41^

Here, we unveil the optical, electronic, and structural properties of 1ML-defined colloidal 2D PbSe fQDs experiencing extreme quantum confinement. By STM we show that while the lateral dimensions of PbSe fQDs remain constant, the thickness of the fQDs varies from 1.8 nm down to a monolayer of cubic PbSe, i.e., 0.6 nm. Meanwhile, the corresponding STS at 77 K unambiguously shows in-gap state free DOS with well-defined electronic gap values of 1.67, 1.26, and 1.00 eV, corresponding to mono-, bi-, and trilayer cubic PbSe, accurately withstanding direct comparison with TB calculations. From these calculations we further predict that the electronic band gap is tunable by more than 0.7 eV by changing the lateral dimensions of the PbSe fQDs from 2 × 2 nm^2^ to 8 × 8 nm^2^. In agreement with STS, three PL contributions are observed in ensemble spectra, with their respective ratios changing for aged samples and suggesting an Ostwald ripening mechanism of thinner (1 and 2 ML) populations of PbSe fQDs toward thicker (2 and 3 ML) ones with a smaller band gap. Strikingly, by comparing the electronic and the optical band gap, we directly obtain the exciton binding energy, which increases from 200 up to 600 meV when the thickness of PbSe fQDs is decreased from 3ML to 1ML. From our experimental results and exciton binding energies for PbSe quantum wells^42^ and quantum wires,^43^ we show that in PbSe nanostructures the exciton binding energy is inversely proportional to the strongest confinement dimension. These findings advance the understanding of colloidal PbSe fQDs by disentangling the vertical and lateral quantum confinement in the ultrathin structures, consequently enabling the synthetic targeting of specific fQD dimensions, leading to tailored optical properties at technologically relevant wavelengths.

NIR emitting colloidal PbSe fQDs are synthesized by a method described by us previously and stabilized by oleate and octylamine ligands.^31,33^ Figure 1a shows an overview HR-HAADF-STEM image of PbSe fQDs exhibiting shapes with slightly anisotropic lateral dimensions of (4.9 ± 1.1) × (3.9 ± 0.6) nm^2^ and a corresponding small aspect ratio of 1.25:1 (see Figure S1a–c). At higher magnification (Figure 1b), the lattice fringes of PbSe fQDs are clearly visible, with the measured lattice spacing of 3.1 Å (200) indicating the PbSe rock salt structure. This is supported by the corresponding FFT pattern (Figure 1c), which exhibits a set of diffraction peaks characteristic of cubic PbSe (lattice constant *a* = 6.128 Å, space group *Fm*3̅*m*, PDF card 01-077-0245). For complete structural characterization, we perform STM imaging to determine the thickness of the PbSe fQDs. Figure 1d depicts a large-scale STM image (23 × 23 nm^2^ at *T* = 77 K) of the sample shown in Figure 1a–c. The size and the morphology of fQDs in STM (Figure 1d,e and Figure S1d–f) are identical to those obtained under STEM (see Figure S1a–c) and show that fQDs are stable under the STM imaging environment without undergoing surface reconstruction. For isolated PbSe fQDs, the height, *z*, can be determined with an accuracy of ∼0.1 nm from STM images collected in constant current mode. Figure 1f shows that, while the lateral dimension remains constant across the sample, three distinct thicknesses of 1.8, 1.2, and 0.6 nm are observed. It is noteworthy that during our experiments we have mostly observed 0.6 or 1.2 nm thick PbSe fQDs and only a few examples with a thickness of 1.8 nm. We attribute the measured thicknesses of 0.6, 1.2, and 1.8 nm to monolayer, bilayer, and trilayer fQDs, respectively, with 0.6 nm being the height of the unit cell of cubic PbSe (Pb-to-Pb distance).

**Figure 1:**
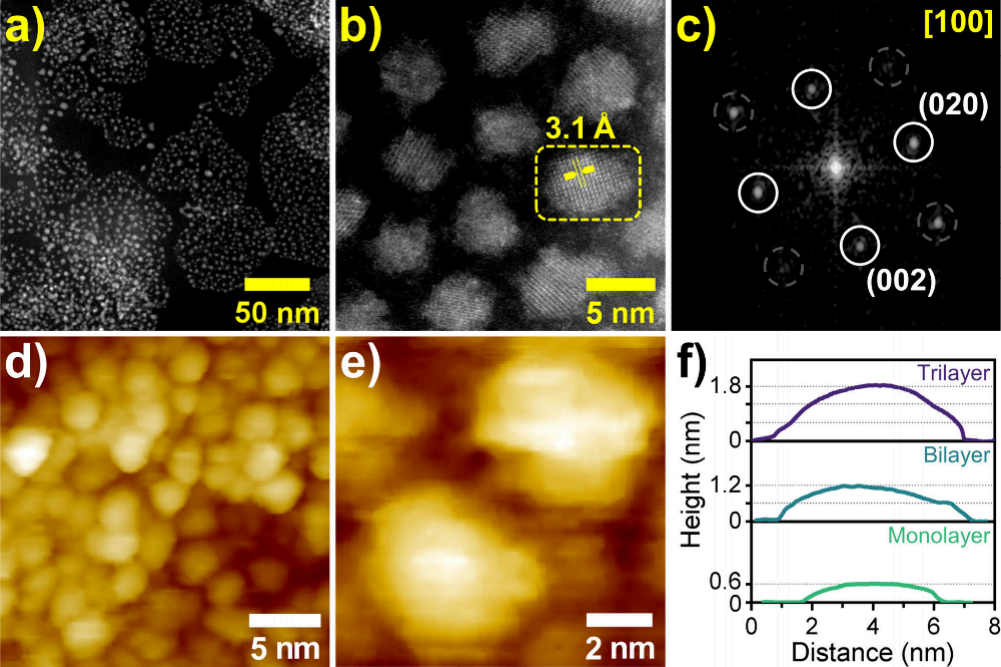
(a) Overview HR-HAADF-STEM image of PbSe fQDs with average lateral dimensions of (4.9 ± 1.1) × (3.9 ± 0.6) nm^2^. (b) High-resolution micrograph of individual crystalline PbSe fQDs (lattice spacing 3.1 Å (200)). (c) FFT pattern of the single fQD region marked in (b), underpinning the crystallinity of the fQD and exhibiting the characteristic diffraction peaks of cubic rock salt structure PbSe. (d) STM image (*V*_S_ = 4 V; *I*_set_ = 50 pA) of the PbSe fQDs shown in (a), exhibiting average lateral sizes of (5.0 ± 1.7) × (3.5 ± 1.3) nm^2^ after annealing under high vacuum. (e) Small-scale STM image (*V*_S_ = 3 V; *I*_set_ = 40 pA) of two PbSe fQDs. (f) STM height profiles of fQDs with thicknesses of 1.8, 1.2, and 0.6 nm.

To gain further insight into the electronic properties of colloidal mono- to few-layer PbSe, we performed STS measurements on isolated individual fQDs at 77 K (Figure 2). STS-measured d*I*/d*V* spectra depend on the parameters of the double barrier tunnel junction formed between the STS tip, the sample, and the conductive substrate. In order to extract valid energy level structures and band gaps, resonant tunneling is required (shell-tunneling regime).^35,44,45^ Figure 2a shows current-dependent d*I*/d*V* spectra collected on a thin PbSe fQD; varying the set-point current *I*_set_ changes the tip to fQD distance and, thereby, the tunneling rate. When *I*_set_ is increased from 100 to 500 pA, the apparent band gap remains constant, which implies a single tunneling junction, i.e., between the tip and the PbSe fQD, which prevents considerable charging of the sample. Please note that the lack of a double tunnel junction points toward an absence of ligands between the fQD and the gold substrate, in agreement with the height measurement by STM. A constant apparent band gap further implies resonant tunneling and that the peaks align with the actual energy levels of the PbSe fQDs, from which we can derive meaningful band gap values. In agreement with Overgaag et al.,^36^ we attribute the tolerance of PbSe fQDs to high set-point currents to the low effective charge carrier masses of PbSe, which result in spatially drawn-out orbitals, and a large tunneling coupling. For the PbSe fQD shown in the STM image inset in Figure 2b, we find a zero-conductance region free of in-gap states, framed by single pronounced peaks on either side. The presence of sharp resonances in the d*I*/d*V* spectra, rather than a step-like energy level structure that would be expected for “truly” 2D quantum materials, provides evidence for the quantum confinement of 2D PbSe in all three dimensions (*x*,*y*,*z*/*a*_B_ < 1) and justifies the use of the term “flat QD”. In accordance with the literature, we assign the two peaks at negative and positive sample voltages to the 1*S*_h_ and 1*S*_e_ energy levels, respectively.^37,46^ The extracted electronic band gap of 1.65 eV is approximately 0.45 eV wider than the largest STS band gap *E*_g, STS_ reported for spherical PbSe QDs (*E*_g, STS,spherical PbSe QDs_ ≈ 1.2 eV for *d* ≈ 3.5 nm) by Liljeroth et al. and highlights the extreme additional confinement in the *z* direction in PbSe fQDs. Figure 2b depicts d*I*/d*V* spectra obtained from four randomly selected spots within the same single PbSe fQD (shown in the inset). Although the expression of the valence (VB) and conduction band (CB) energy levels varies slightly between the different spots, we do not find in-gap states at any of the probed spots. Furthermore, the measured band gap value is consistent throughout the fQD, underpinning that the deduced *E*_g, STS_ values are valid.

**Figure 2:**
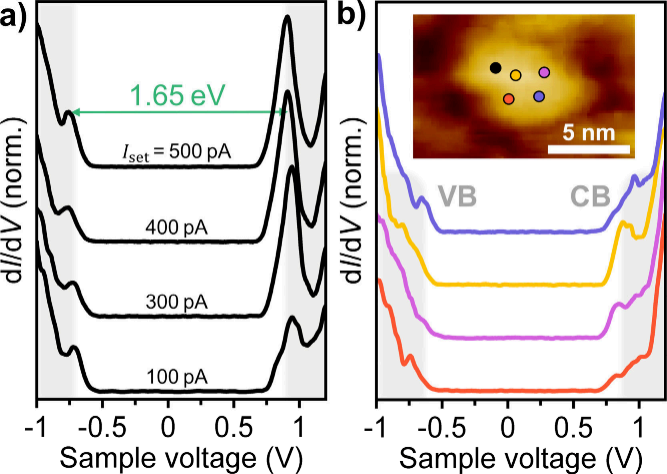
(a) Set-point current dependent STS spectra on the black dot area of the PbSe fQD (*E*_g, STS_ = 1.65 eV) shown in the inset of panel b. (b) d*I*/d*V* spectra (*V*_S_ = −1 V; *I*_set_ = 200 pA) measured at four different spots on the same PbSe fQD (color-coded in the STM image inset), which consistently show a band gap free of in-gap states with only minor fluctuations in the form of the VB and CB. Inset: STM image (*V*_S_ = 4 V; *I*_set_ = 50 pA) of the fQD.

Figure 3a shows representative d*I*/d*V* spectra at *T* = 77 K for PbSe fQDs with varying thickness. The electronic band gap increases from 1.00 to 1.67 eV when the thickness of the PbSe fQDs is decreased from 1.8 to 0.6 nm as a result of the strong quantum confinement. To further understand the correlation between the thickness/layer number and the band gap size, we performed TB calculations (Figure 3b) for PbSe fQDs with lateral dimensions of 4 × 4 nm^2^.^47^ The theoretical DOS quantitatively replicates the DOS and the electronic band gap derived by STS for mono-, bi-, and trilayer thick PbSe fQDs, confirming the STM height measurement shown in Figure 1f spectroscopically. Figure 3c depicts a schematic visualization of the crystal structure of a monolayer, bilayer, and trilayer cubic rock salt PbSe fQD (the crystal was built using the open-source molecular builder and visualization tool Avogadro version 1.2.0 with library version 1.2.0 ^48^). The weak variations between the calculated and experimental electronic gaps likely arise from the slight deviation from a square shape of the fQDs.

**Figure 3:**
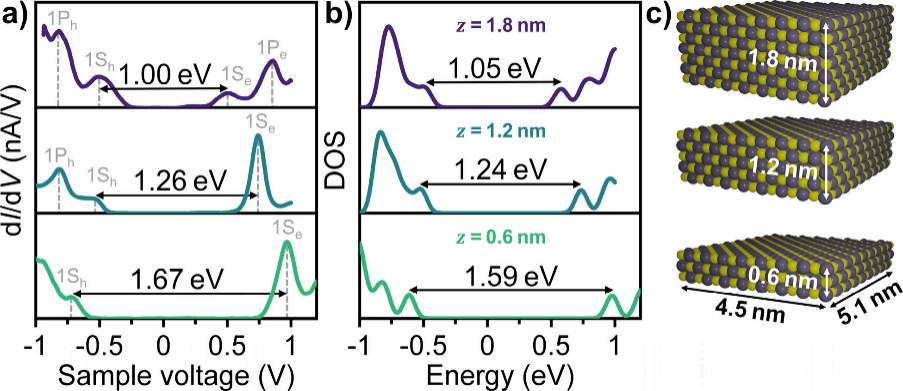
(a) Representative STS spectra at 77 K of fQDs with tri-, bi-, and monolayer thickness (top to bottom; tunneling conditions: *V*_s_ = −1 V, *I*_set_ = 200 pA). (b) Theoretical TB calculated DOS of PbSe fQDs with three different thicknesses *z*. Note that a broadening of 60 meV is introduced to the calculations to reproduce the experimental conditions. (c) Schematic representation of a monolayer, bilayer, and trilayer cubic rock salt PbSe fQD of 5.1 × 4.5 nm^2^ with atomically flat (001) basal and site facets, with alternating lead (gray) and selenium (yellow) atoms.

In addition to their remarkable structural and electronic properties, PbSe fQDs are also appealing with regard to their optical properties. Figure 4a shows the RT ensemble PL and absorbance spectrum of the PbSe fQD sample investigated by STM/S (sample 1) directly after the synthesis. The PL is centered near 0.94 eV (fwhm = 197 meV) with weakly pronounced excitonic absorption at 1.23 eV. Surprisingly, cryogenic ensemble PL measurements performed 15 months after the STM/S measurements (Figure 4a) reveal a rather narrow PL line centered at 0.80 eV (1550 nm; fwhm = 120 meV) in the C-band telecommunication window. We infer Ostwald ripening of PbSe fQD 1MLs and bilayers toward trilayer fQDs in the metastable colloidal solution over the extended time frame as the reason for the bathochromic PL shift (compared to the PL directly after synthesis) and the PL being governed by the emission of the thicker PbSe fQDs (see Figure S2 and Table S1 for further discussion on Ostwald ripening/fusing of fQDs). To test this hypothesis, we synthesized a new batch of PbSe fQDs (sample 2) with a smaller lateral size (3.3 ± 0.5) × (2.9 ± 0.4) nm^2^ (see Figure S3), exhibiting optical features at higher energies for the following cryogenic PL discussion (Figure 4b). When investigating a fQD sample directly after synthesis, we find that the cryogenic PL spectrum is best fitted by the sum of three Gaussians, which reflects the three distinct fQD thickness populations (1ML (0.6 nm), bilayer (1.2 nm), and trilayer (1.8 nm)) and points to an absence of major Ostwald ripening (Figure 4b). The fwhm of each spectral feature at cryogenic temperature in Figure 4a and b is comparable to the values typically obtained for spherical PbSe QDs (50–100 meV)^2,20^ (although it should be noted that the fwhm of 2D PbSe^31,33^ strongly depends on the spectral position and is smaller the further the PL lies in the NIR), and all contributions are significantly narrower than typical values for comparable 2D PbS nanoplatelets at RT (>200 meV),^21,34^ indicating the potential of PbSe fQDs for applications requiring narrow emission.

**Figure 4:**
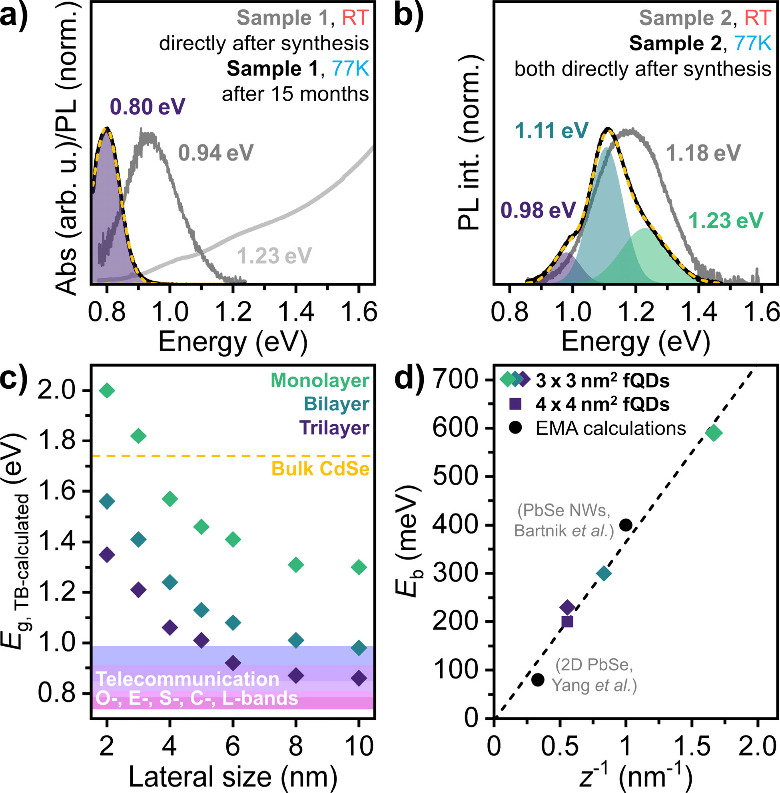
(a) Ensemble RT absorbance and PL spectrum (gray) of the colloidal PbSe fQDs studied in STM/S and cryogenic PL spectrum of the same sample over a year later (black), showing bathochromically shifted narrow PL in the third telecommunication window at 0.80 eV (1550 nm), which is attributed to Ostwald ripening within the time frame. (b) Cryogenic PL spectrum of laterally smaller PbSe fQDs measured directly after synthesis. The three PL contributions are attributed to monolayer, bilayer, and trilayer PbSe fQD populations. (c) TB calculated correlation between the electronic band gap and lateral size of mono- to trilayer PbSe fQDs, highlighting the interplay of strong vertical confinement and the minor lateral confinement in pushing PbSe fQD emission into the low attenuation telecommunication windows. (d) Exciton binding energy vs the reciprocal value of the thickness *z* of PbSe fQDs from this work and 2D PbSe^42^ as well as PbSe nanowires (NWs)^43^ (both calculated by effective mass approximation (EMA)).

We further performed TB calculations to determine the influence of the lateral size on quantum confinement in PbSe fQDs. Figure 4c shows the electronic band gap deduced from the calculated DOS (see Figure S4 for the calculated DOS corresponding to each of the plotted data points) for square-shaped fQDs with a thickness from mono- to trilayers and lateral dimensions ranging from 2 to 10 nm. For each fQD thickness, the electronic gap dramatically drops by 600 meV when tuning the size from 2 to 6 nm. Beyond 6 nm, the electronic band gap weakly changes and the quantum confinement is compelled by the thickness of the fQDs. This substantiates previous reports on the PL tunability of ultrathin PbSe^31^ and PbTe^32^ NPLs, which was assumed to be caused by a combination of the thickness **and** the lateral size, with thickness as the major factor. In addition, Figure 4c highlights the possibility and provides a target range to access PL in the low attenuation telecommunication range of the O-, E-, and S-bands, where glass fibers exhibit negligible losses, by further tailoring the dimensions of PbSe fQDs.

It is noteworthy that the exciton binding energy of PbSe fQDs can be directly determined by our experimental results. It corresponds to the difference between the experimental values of the electronic and optical band gap with the latter including electron–hole Coulomb interactions. For the sample shown in Figure 4b, we evaluate the exciton binding energy as the difference between the electronic gap derived from TB calculations for 3 × 3 nm^2^ and the optical spectra. We find that the binding energy *E*_b_ increases from 200 meV for 1.8 nm thick PbSe fQDs up to a record value of 600 meV for PbSe 1MLs, which is comparable to well-established layered materials such as transition metal dichalcogenides, for which *E*_b_ typically exhibits values of ∼0.5 eV.^49^ We emphasize that our experimental results validate previous estimations from 4-band effective mass calculations on the exciton binding energy in low-dimensional PbSe nanomaterials. Figure 4d shows our experimental exciton binding energies together with the ones estimated by EMA calculations for 3 nm thick PbSe nanosheets (*E*_b_ ∼ 80 meV)^42^ and ultranarrow (1 nm in diameter) PbSe NWs (*E*_b_ ∼ 400 meV).^43^ The exciton binding energy is inversely proportional to the dimension that compels the quantum confinement.

In conclusion, we have synthesized NIR-emitting colloidal PbSe fQDs with thicknesses down to the monolayer. We directly demonstrate the strongly anisotropic nature of the fQDs by STM (height) measurements and confirm the microscopy data by a comprehensive study of the electronic properties via low-temperature STS and TB calculations. In-gap state free electronic band gaps of 1.67 1.26, and 1.00 eV for mono-, bi-, and trilayers and a QD-like DOS with defined peaks on either side of the zero-conductance region, in excellent agreement with theoretical DOS, are demonstrated. Associated ensemble cryogenic PL spectra contain three distinct contributions, which are attributed to the different thickness populations in PbSe fQD syntheses and show consistent results between structural, electronic, and optical properties. From a shift of the PL contributions on longer time scales after synthesis, we infer Ostwald ripening and a possible fusing mechanism of thinner fQDs to thicker populations with PL shifting to longer telecommunication wavelengths, which can be slowed down by PbI_2_ surface passivation. Our study showcases the extreme quantum confinement of PbSe fQDs exhibiting high exciton binding energies up to 600 meV for PbSe 1MLs and emphasizes their potential as innovative classical or quantum light sources for fiber optics-based photonics.

## Supplementary Material



## Notes

Typical TEM images used for determining lateral PbSe fQD dimensions; size histogram of PbSe fQDs from TEM; typical STM images used for determining lateral PbSe fQDs dimensions; size histogram of PbSe fQDs from STM; cryogenic PL spectra of pristine and PbI_2_-passivated PbSe fQDs and a discussion on Ostwald ripening; position and ratio of all PL contributions shown in PL spectra in the manuscript and the SI; exemplary TEM image and lateral size histogram of laterally smaller PbSe fQDs; TB-calculated DOS of mono- to trilayer PbSe fQDs with different lateral sizes (PDF)

## ABBREVIATIONS

0D: zero-dimensional
2D: two-dimensional
CB: conduction band
DOS: density of states
EMA: effective mass approximation
FFT: fast Fourier transform
fQD: flat quantum dot
fwhm: full width at half-maximum
HR-HAADF-STEM: high-resolution high-angle annular dark-field scanning transmission microscopy
1ML: monolayer
NC: nanocrystal
NW: nanowire
NIR: near-infrared
NPL: nanoplatelet
PDF: powder diffraction file
PL: photoluminescence
QD: quantum dot
QY: quantum yield
RT: room temperature
STM: scanning tunneling microscopy
STS: scanning tunneling spectroscopy
TB: tight-binding
VB: valence band
