# Supplementary material for: Monolayer-Defined Flat Colloidal PbSe Quantum Dots in Extreme Confinement
Source: Nano Lett. 2025 Jul 28;25(31):12019–24. doi: 10.1021/acs.nanolett.5c02957 (PMC12333419; doi:10.1021/acs.nanolett.5c02957)
Supplement: Supplementary file 1 [file nl5c02957_si_001.pdf]

Supporting Information

Monolayer-Defined Flat Colloidal PbSe Quantum Dots in Extreme Confinement

Leon Biesterfeld,[a,b,c] *Huu Thoai Ngo,*[d] *Ahmed Addad,*[e] *Dominik A. Rudolph,*[a,c] *Wolfgang Leis,*[f] *Michael Seitz,*[f] *Gang Ji,*[e] *Bruno Grandidier,*[d] *Christophe Delerue,*[d] *Jannika Lauth,* *[a,b,c] *Louis Biadala* *[d]

a – Cluster of Excellence PhoenixD (Photonics, Optics, and Engineering – Innovation Across Disciplines), Welfengarten 1A, D-30167 Hannover, Germany.

b – Institute of Physical and Theoretical Chemistry, Eberhard Karls University of Tübingen, Auf der Morgenstelle 18, D-72076 Tübingen, Germany.

c – Institute of Physical Chemistry and Electrochemistry, Leibniz University Hannover, Callinstr. 3A, D-30167 Hannover, Germany.

d – Université de Lille, CNRS, Centrale Lille, Université Polytechnique Hauts-de-France, Junia-ISEN, UMR 8520-IEMN, F-59000 Lille, France.

e – Université Lille, CNRS, INRAE, Centrale Lille, UMR 8207-UMET-Unité Matériaux et Transformations, F-59000 Lille, France.

f – Institute of Inorganic Chemistry, Eberhard Karls University of Tübingen, Auf der Morgenstelle 18, D-72076 Tübingen, Germany.

*louis.biadala@iemn.fr and jannika.lauth@uni-tuebingen.de

Supplementary Notes

Chemicals. Acetonitrile (≥ 99.5 %), anhydrous ethanol (max. 0.01 % H_2O), isopropanol (≥ 99.5 %), lead(II) oxide (≥ 99.99 %), methanol (≥ 99.8 %), *n*-octylamine (99 %), anhydrous tetrachloroethylene (TCE, ≥ 99 %), triethylamine (≥ 99 %), trifluoroacetic acid (99 %), and trifluoroacetic anhydride (≥ 99 %) were purchased from Sigma-Aldrich/Merck. Anhydrous *n*-hexane (97 %) was purchased from Acros Organics. Lead(II) iodide (99.99 %) and selenourea (99.97 %) were purchased from Alfa Aesar. Oleic acid (90 %) was purchased from ABCR. *n*-Octylamine and oleic acid were degassed *via* the freeze-pump-thaw technique three times prior to being used and stored inside a N_2-filled glovebox. All other reagents were used as received from the listed suppliers without any further purification steps.

PbSe fQDs Synthesis. 2D PbSe fQDs were synthesized following a procedure previously described by our group;[1,2] all synthetic steps were performed under N_2 atmosphere. Prior to the actual synthesis, a selenourea precursor solution was prepared by dissolving selenourea (193 mg, 1.57 mmol) in a mixture of octylamine (2.025 ml, 12.2 mmol), oleic acid (0.225 ml, 0.71 mmol), and *n*-hexane (0.75 ml) at 35°C for 24 - 72 h. For a typical 2D PbSe fQDs synthesis, lead oleate

(1.83 g, 2.7 mmol) (synthesized *via* an established method of Hendricks *et al.*)[3] was stirred in a mixture of octylamine (2.0 ml, 12.0 mmol), oleic acid (4.0 ml, 12.5 mmol), and *n*-hexane (18 ml) at 35°C until complete dissolution and subsequently cooled to 0°C using an ice bath. The selenourea solution (2.5 ml) was injected rapidly into the vigorously stirred lead oleate solution, causing an immediate color change from colorless/slightly yellow to dark brown. After 10 min the dark reaction mixture was quenched by adding dry ethanol (18.5 ml). The cloudy quenched solution was then centrifuged at 2500 rcf for 10 min, the supernatant discarded, and the precipitate was redispersed in *n*-hexane (10 ml). This process was repeated until the supernatant was transparent, usually requiring a total of three cycles. At this point, pristine PbSe fQDs were sealed under N_2 atmosphere and stored in a refrigerator. PbI_2 passivated PbSe fQDs were prepared by diluting pristine fQDs (0.5 ml) with *n*-hexane (0.75 ml) and injecting 1.0 ml of a 0.1 M PbI_2 solution in *n*-octylamine and oleic acid (molar ratio 9:1). After 40 min at 35°C the passivated PbSe fQDs were likewise sealed and stored in a refrigerator.

High-Angle Annular Dark-Field Scanning Transmission Electron Microscopy. HR-HAADF-STEM images were obtained using a FEI Titan Themis 300 microscope equipped with a probe aberration corrector, operated at 200 kV. The probe size was set to 0.1 nm with a convergence semi-angle of 22.5 mrad. The collection angle of the HAADF detector was in the range of 80 - 150 mrad. The contrast in HAADF images is proportional to $Z \approx 1.7 - 2$, meaning that bright contrast indicates relatively heavy atomic composition.

Transmission Electron Microscopy. TEM images were acquired using a FEI Tecnai G2 F20 transmission electron microscope with a field emission gun operating at 200 kV. For TEM measurements the colloidal PbSe fQDs were crop-cast onto carbon-coated copper grids (300 mesh) from Quantifoil.

Scanning Tunneling Microscopy and Spectroscopy. Colloidal solutions of PbSe fQDs were drop-cast onto gold substrates, which were immediately loaded into the load lock chamber of a ultra-high vacuum (UHV) system (base pressure below 1 x 10^{-10} Torr) for annealing at 80°C for several hours. The samples were probed with tungsten tips that were thoroughly annealed in UHV. The STM experiments were performed with a low temperature STM from Omicron at 77 K. The STM images were typically obtained with a sample bias between -5.5 and -4.5 V and set-point currents less than 30 pA. Tunneling spectroscopy measurements were performed at constant tip-sample separation by varying the feedback bias between -2.0 and -1.0 V and increasing the set-point current between hundreds of pA. The dynamic conductance was measured with a lock-in amplifier typically using a 9 mV modulation at 500 Hz on the bias voltage. During the acquisition of a sequence of spectra, ramping up the voltage between the sample and the tip took 4.6 s to measure a single $\mathrm{d}I/\mathrm{d}V$ trace.

NIR (Cryogenic) PL and Ultraviolet-Visible-NIR Absorbance Spectroscopy. NIR (cryogenic) PL spectra were collected using an Edinburgh FLS 1000 UV-Vis-NIR spectrometer and a PTI QuantaMaster QM4 spectrofluorometer. The Edinburgh FLS 1000 is equipped with a 450 W ozone-free xenon arc lamp for excitation; PL was monitored using a liquid N_2 cooled InGaAs NIR photomultiplier tube 1650 detector from Edinburgh. The PTI QuantaMaster QM4 is equipped with a 75 W steady-state xenon short arc lamp for excitation and PL was monitored using a liquid N_2 cooled PTI P1.7R detector module (Hamamatsu PMT R5509-72). To avoid higher order excitation light, a RG780 long pass filter glass (thickness of 3 mm) was mounted in the emission path. Spectral selection was achieved using single grating monochromators (excitation: 1200 grooves/mm, 300 nm blaze; NIR emission 600 grooves/mm, 1200 nm blaze). Samples for RT optical ensemble spectroscopy were prepared by diluting the colloidal PbSe fQD solutions in

TCE (optical density below 0.2 at 500 nm) in a quartz cuvette (quartz glass high performance QS 200 - 2500 nm with an optical path length of 1 cm by Hellma). Absolute PLQYs were determined with the FLS 1000 using an integrating sphere. For this, scattering at 450 nm and the PL in the NIR of TCE and the fQDs were measured separately, accounting for the difference in sensitivity of both detectors with a correction factor. Cryogenic PL spectra measured with the FLS 1000 were acquired using an Optistat CF sample-in-vacuum cryostat system by Oxford Instruments operated with liquid N_2 at regulated temperatures between 293 K and 77 K. Samples were prepared by drop-casting the colloidal PbSe fQD solutions onto high purity fused silica substrates from Esco Optics. Cryogenic PL spectra obtained with the QuantaMaster QM4 were collected using a special liquid N_2 filled dewar accessory with a fused silica base at the level of the optical path. For this, samples were prepared by dip-coating magnesia sticks (from Sigma-Aldrich/Merck) in the colloidal PbSe fQD solutions, which were then directly submerged in the liquid N_2, achieving operating temperatures of ~ 80 K. All PL spectra were acquired by exciting the PbSe fQDs at 450 nm. UV-Vis-NIR absorbance spectra were collected by using a double beam Cary 5000 spectrophotometer from Agilent Technologies equipped with a tungsten halogen (Vis) and deuterium arc (UV) lamp and a PbSmart NIR detector for monitoring.

Tight-Binding Calculations. We calculate the electronic structure of QDs in TB. Pb and Se atoms are described by 20 atomic orbitals, 10 orbitals ($sp^3d^5s^*$) for each spin. The Hamiltonian matrix is written from parameters that have been adjusted to give a good description of the electronic structure of PbSe over a wide energy range and the entire Brillouin zone. Spin-orbit coupling is included. We present here the results obtained with parameters by Poddubny *et al.*,[4] but very similar results are obtained with parameters by Allan *et al.*[5] Due to the large number of atoms in QDs, full diagonalization of the Hamiltonian is impossible, and only electronic states

close to the gap are calculated. The DOS is obtained by expanding each Dirac by a Gaussian with a half-value width of 100 meV. QD surfaces are not passivated. Despite this, in the case of IV-VI semiconductors, there are no surface states for reasons discussed in Ref. 5, which was confirmed by *ab initio* calculations by Hens *et al.*[6]

Supplementary Figures and Tables

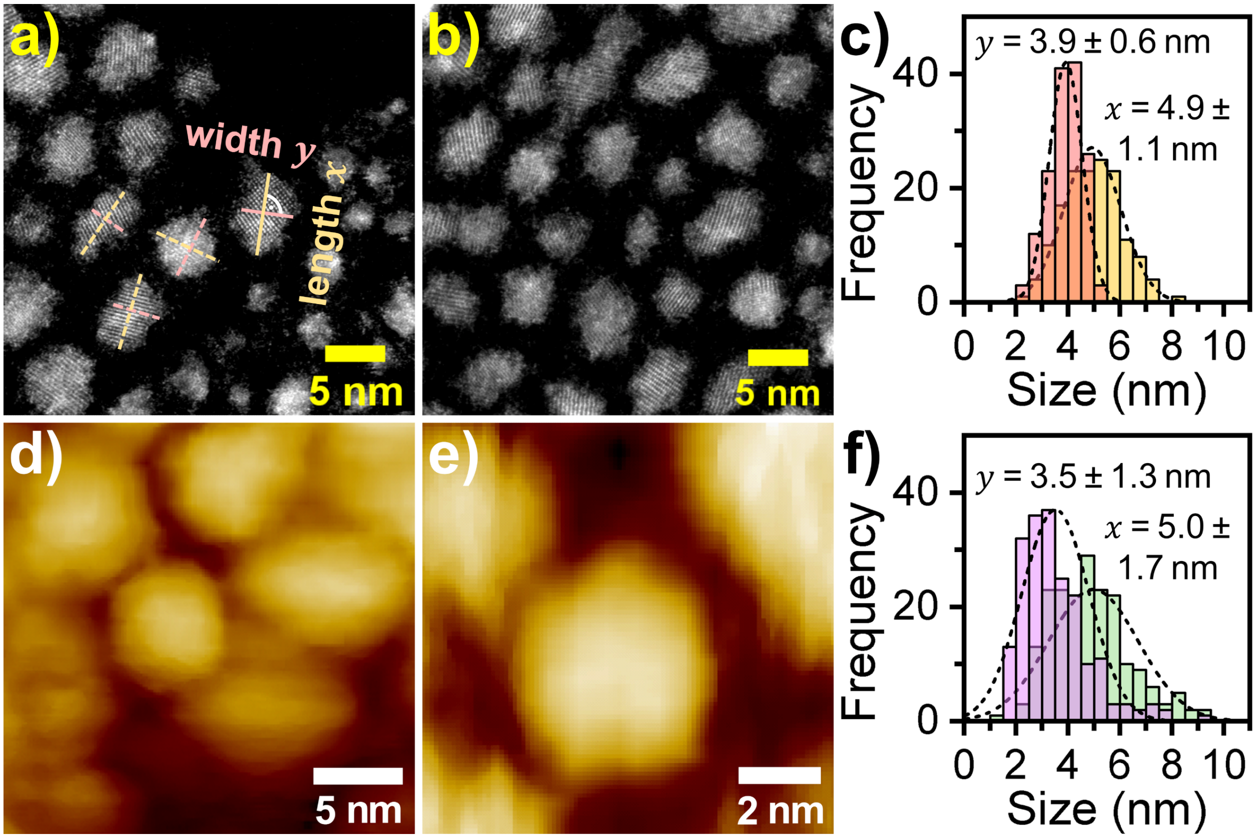

Figure S1. (a, b) Typical STEM images used for lateral size determination of PbSe fQDs. x-Lengths were determined by measuring the longest dimension of the NPLs, y-widths are the largest distance orthogonal to the x-length. (c) Lateral size histogram of PbSe fQDs, values determined from STEM images. (d, e) Typical STM images used for lateral size determination after annealing under high vacuum. (f) Lateral size histogram of PbSe fQDs determined from STM images, demonstrating that the size of the fQDs does not change drastically during annealing.

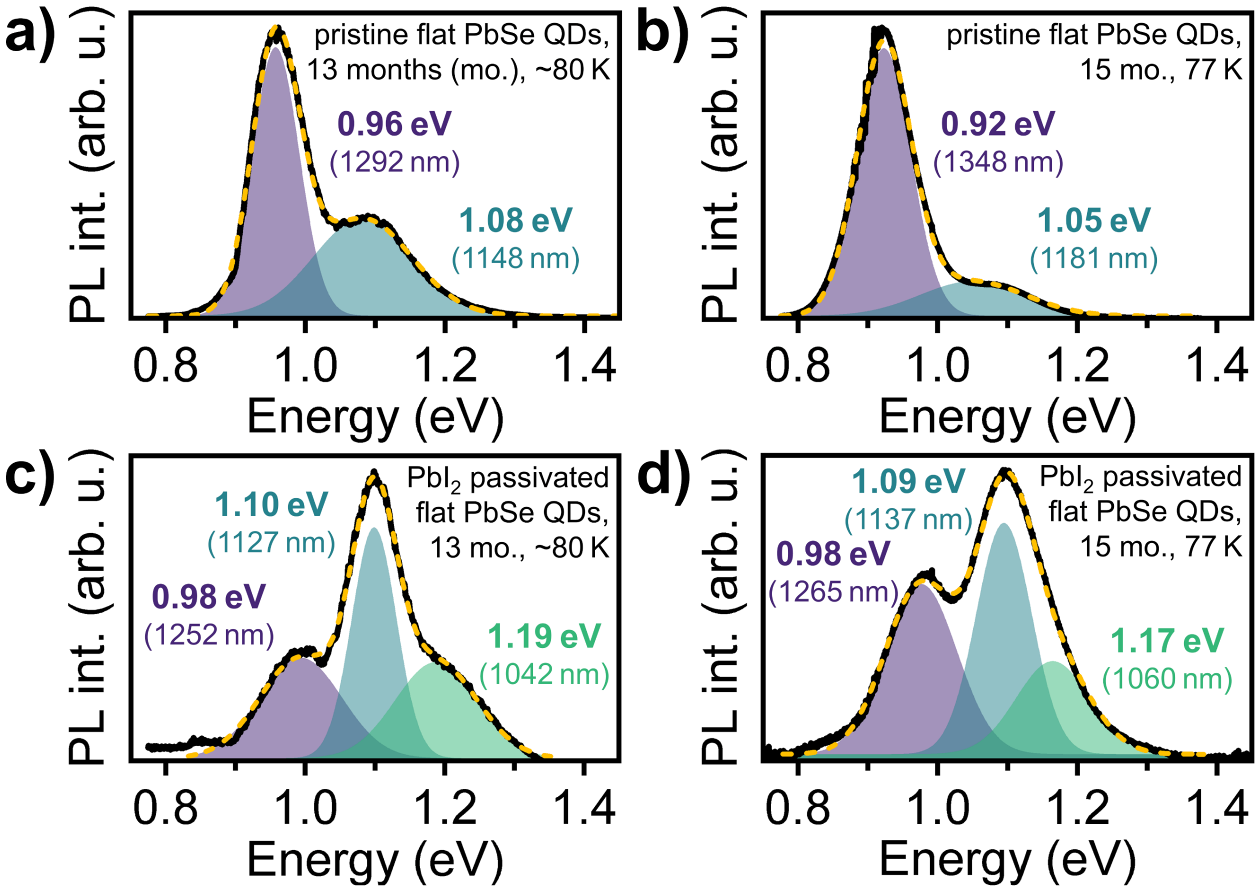

Figure S2. Cryogenic PL spectra of pristine (a, b) and PbI_2 passivated (c, d) PbSe fQDs,[1] measured two months apart (during this time the samples were stored at ambient temperature under N_2 atmosphere). (a, b) The low temperature PL of pristine fQDs is best fitted by the sum of two Gaussians, assigned to bi- and trilayer PbSe, resp. (c, d) For PbI_2 passivated PbSe fQDs, the PL is best fitted by three Gaussians in both cases. The third and highest energy PL contribution is attributed to 1ML PbSe fQDs. Notably, the ratio between the two/three PL contributions changes within the studied time frame; after two months, we observe an increased population of the lower energy contributions (bilayer and trilayer PbSe fQDs), while the higher energy contributions (1ML and bilayer PbSe fQDs) decrease (see Table S1 for details on PL contributions of all samples shown in the main manuscript and the SI). As stated in the main manuscript, we infer Ostwald ripening in solution (and fusing in deposited samples) as the cause of shifted PL maxima in aged

fQD samples and the ratio changes of the three contributions. In particular, we observe more pronounced ageing in pristine PbSe fQDs (a, b) with a surface mainly passivated by oleate in octylamine as a solvent (no 1ML contribution after 13 and 15 months, compared to the new sample 2 shown in Figure 4b of the main manuscript, which shows all three contributions). In contrast, PbI_2 passivation appears to be a valuable tool for slowing down/hindering the fusing of fQDs (c, d) and we find three PL contributions at both time points. These observations are consistent with previous reports for 2D CdSe NPLs by Dufour *et al.*,[7] who reported an increased colloidal stability for mixed halide- and octylamine-passivated 2D CdSe NPLs compared to purely organically passivated NPLs with a tendency to stack over time. Directly related to PbSe, Koh *et al.* found that oriented attachment of PbSe QDs is disfavored when PbI_2/I^- is introduced, since the mismatch between Pb-I and Pb-Se bond lengths prevents the formation of bridges between adjacent NCs,[8] which likewise could hinder the vertical fusing of fQDs in our case. Similarly, we have previously reported the necessity of PbI_2 passivation for the colloidal stabilization of 2D PbTe NPLs synthesized under comparable conditions.[9]

Table S1. Position and ratio of all PL contributions for the PL spectra shown in Figure 4a and b in the main manuscript and Figure S2.

**Sample**	**1ML PL contribution**		**Bilayer PL contribution**		**Trilayer PL contribution**	
	**Position (eV)**	**Ratio (%)**	**Position (eV)**	**Ratio (%)**	**Position (eV)**	**Ratio (%)**
STM/S sample, 77 K, after 15 mo. (Figure 4a)	-	-	-	-	0.80	100
Laterally smaller PbSe fQDs, 77 K, directly after synthesis (Figure 4b)	1.23	33	1.11	56	0.98	11
Pristine PbSe fQDs, 13 mo., ~80 K (Figure S2a)	-	-	1.08	43	0.96	57
Pristine PbSe fQDs, 15 mo., 77 K (Figure S2b)	-	-	1.05	20	0.92	80
PbI_2 passivated PbSe fQDs, 13 mo., ~80 K (Figure S2c)	1.19	30	1.10	39	0.98	31
PbI_2 passivated PbSe fQDs, 15 mo., 77 K (Figure S2d)	1.17	22	1.09	40	0.98	38

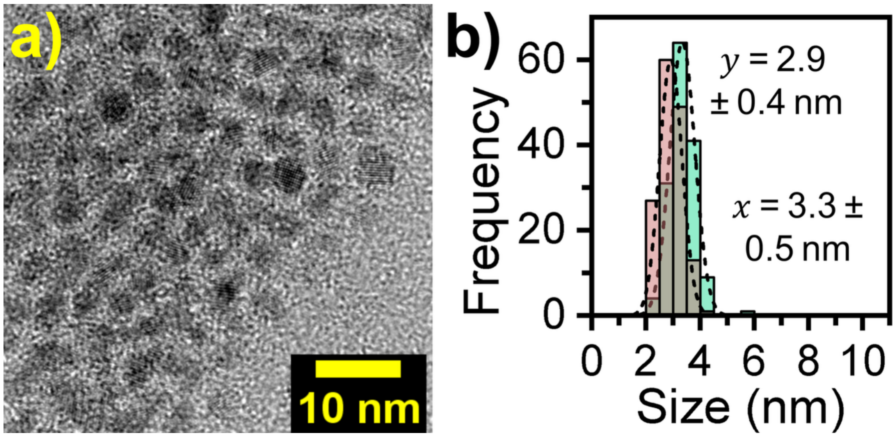

Figure S3. (a) Exemplary TEM image of the laterally smaller PbSe fQDs ((3.3 ± 0.5) x (2.9 ± 0.4) nm^2) shown in Figure 4b of the main manuscript. (b) Corresponding lateral size histogram.

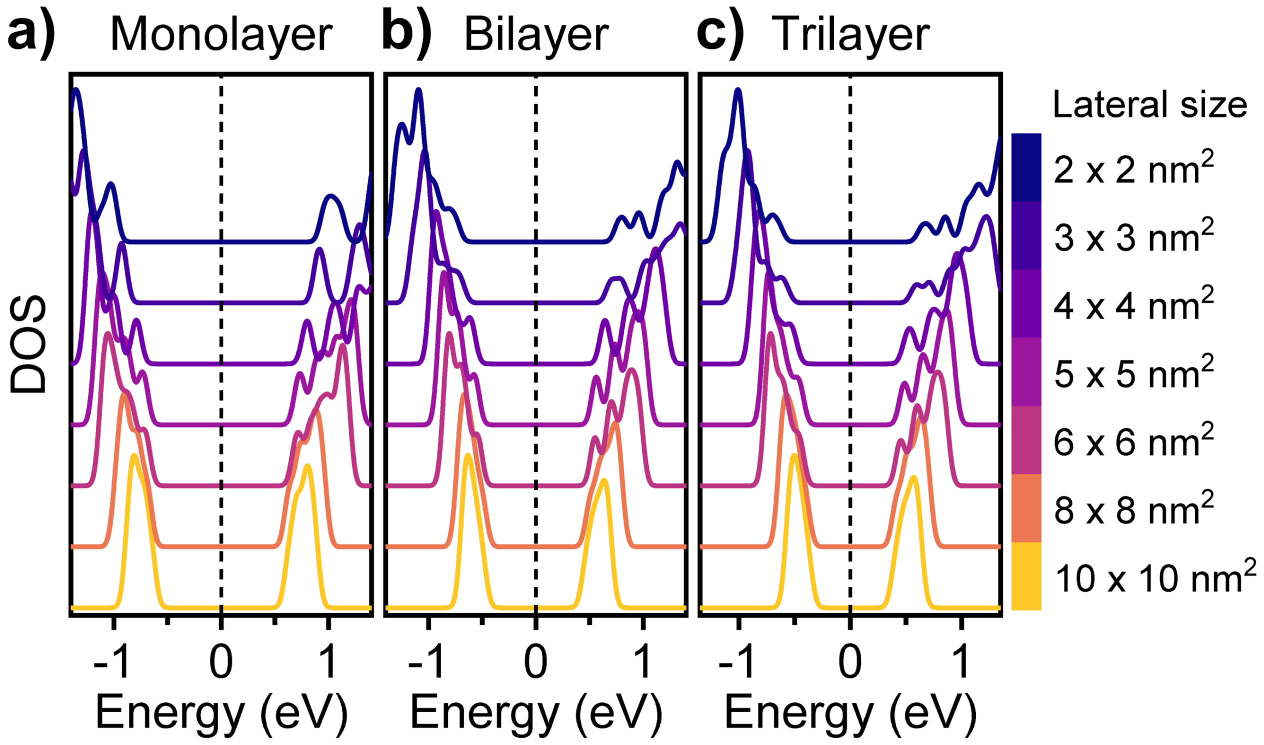

Figure S4. TB calculated DOS for 1ML (a), bilayer (b), and trilayer (c) PbSe fQDs with increasing lateral size from 2 x 2 nm^2 to 10 x 10 nm^2, used to determine the calculated electronic band gaps for the band gap vs. lateral size plot shown in Figure 4c of the main manuscript.

REFERENCES

(1) Biesterfeld, L.; Klepzig, L. F.; Niebur, A.; Rosebrock, M.; Lauth, J. Toward Bright Colloidal Near-Infrared Emitters: Surface Passivation of 2D PbSe Nanoplatelets by Metal Halides. *J. Phys. Chem. C* **2022**, *126* (45), 19277-19285.

(2) Klepzig, L. F.; Biesterfeld, L.; Romain, M.; Niebur, A.; Schlosser, A.; Hübner, J.; Lauth, J. Colloidal 2D PbSe Nanoplatelets with Efficient Emission Reaching the Telecom O-, E- and S-Band. *Nanoscale Adv.* **2022**, *4*, 590-599.

(3) Hendricks, M. P.; Campos, M. P.; Cleveland, G. T.; Jen-La Plante, I.; Owen, J. S. A Tunable Library of Substituted Thiourea Precursors to Metal Sulfide Nanocrystals. *Science* **2015**, *348* (6240), 1226-1230.

(4) Poddubny, A. N.; Nestoklon, M. O.; Goupalov, S. V. Anomalous Suppression of Valley Splittings in Lead Salt Nanocrystals without Inversion Center. *Phys. Rev. B* **2012**, *86* (3), 35324.

(5) Allan, G.; Delerue, C. Confinement Effects in PbSe Quantum Wells and Nanocrystals. *Phys. Rev. B* **2004**, *70* (24), 245321.

(6) Hens, Z.; Llusar, J.; Infante, I. Fuzzy Band Structure of Quantum Dots by Bloch Orbital Expansion: Unconventional Insights into Geometric-Electronic Structure Relations. *ACS Nano* **2025**, *19* (8), 8227-8237.

(7) Dufour, M.; Qu, J.; Greboval, C.; Méthivier, C.; Lhuillier, E.; Ithurria, S. Halide Ligands To Release Strain in Cadmium Chalcogenide Nanoplatelets and Achieve High Brightness. *ACS Nano* **2019**, *13* (5), 5326-5334.

(8) Koh, W.-K.; Dandu, N. K.; Fidler, A. F.; Klimov, V. I.; Pietryga, J. M.; Kilina, S. V. Thickness-Controlled Quasi-Two-Dimensional Colloidal PbSe Nanoplatelets. *J. Am. Chem. Soc.* **2017**, *139* (6), 2152–2155.

(9) Biesterfeld, L.; Vochezer, M. T.; Kögel, M.; Zaluzhnyy, I. A.; Rosebrock, M.; Klepzig, L. F.; Leis, W.; Seitz, M.; Meyer, J. C.; Lauth, J. Solving the Synthetic Riddle of Colloidal Two-Dimensional PbTe Nanoplatelets with Tunable Near-Infrared Emission. *Chem. Mater.* **2024**, *36* (15), 7197-7206.
